# Health losses attributed to anthropogenic climate change

**DOI:** 10.1038/s41558-025-02399-7

**Published:** 2025-09-17

**Authors:** Colin J. Carlson, Dann Mitchell, Rory Gibb, Rupert F. Stuart-Smith, Tamma Carleton, Torre E. Lavelle, Catherine A. Lippi, Megan Lukas-Sithole, Michelle A. North, Sadie J. Ryan, Dorcas Stella Shumba, Matthew Chersich, Mark New, Christopher H. Trisos

**Affiliations:** 1 Yale University School of Public Health, New Haven, CT USA; 2 University of Bristol, Bristol, UK; 3 University College London, London, UK; 4 Oxford Sustainable Law Programme, Smith School of Enterprise and the Environment, University of Oxford, Oxford, UK; 5 University of California Berkeley, Berkeley, CA USA; 6 University of Florida, Gainesville, FL USA; 7 Cape Peninsula University of Technology, Cape Town, South Africa; 8 University of KwaZulu-Natal, Durban, South Africa; 9 African Climate and Development Initiative, University of Cape Town, Rondebosch, South Africa; 10 University of the Witwatersrand, Braamfontein, South Africa; 11 African Synthesis Centre for Environment Climate Change and Development (ASCEND), University of Cape Town, Rondebosch, South Africa

**Keywords:** Attribution, Environmental health, Developing world, Governance

## Abstract

Over the last decade, attribution science has shown that climate change is responsible for substantial death, disability and illness. However, health impact attribution studies have focused disproportionately on populations in high-income countries, and have mostly quantified the health outcomes of heat and extreme weather. A clearer picture of the global burden of climate change could encourage policymakers to treat the climate crisis like a public health emergency.

## Abstract (web summary)

The authors assess the growing field of climate change health impact attribution. They show literature bias towards direct heat effects and extreme weather in high-income countries, highlighting the lack of global representation in current efforts.

## Main

Scientists agree that recent climate change is outside the realm of normal natural variability, that natural factors in the earth system cannot explain the observed changes and that anthropogenic influences are responsible for, at the time of writing^1^, roughly +1.3 °C of global warming from preindustrial levels. They have developed this consensus through a set of quantitative methods that are grouped under the joint umbrella of climate change detection (showing that the climate has changed) and attribution (distinguishing the relative contributions of both anthropogenic and natural influences on the global climate system). In the past decade, researchers have also started using the same methods to isolate the effects of anthropogenic forcings on the social and ecological consequences of climate change. These end-to-end impact attribution studies^2^ account for a small fraction of total research on climate change impacts, but represent some of the strongest evidence in terms of both methodological rigour and ability to articulate clear, quantitative estimates of historical and present-day impacts.

**Fig. 1:**
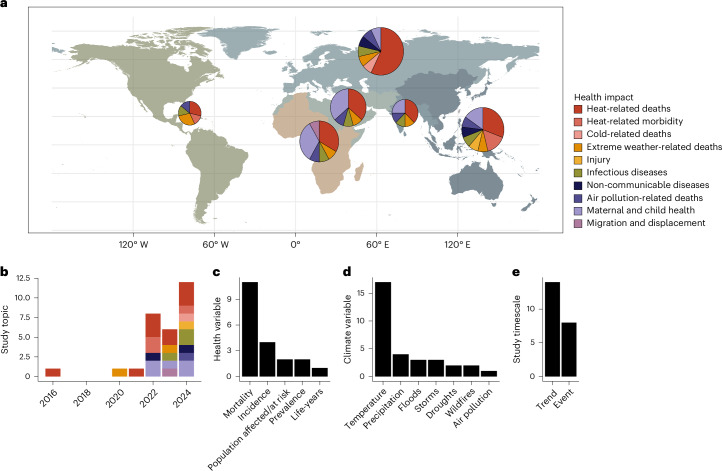
The rise of health impact attribution. **a**, The distribution of research effort by health impact and geography of the affected population. The map is divided by World Health Organization global regions. Circle size is proportional to the number of applicable studies focused on each region, ranging from 7 in the Americas to 14 in Europe. **b**–**e**, The distribution of studies across years (**b**) and study focus (**c**–**e**). Some studies are in multiple categories.

Human health—especially loss of life, but also illness, disability and poor well-being—is one of the most visible categories of climate change impacts. However, most work on the health impacts of climate change has stopped a step short of end-to-end attribution, focusing on long-term trends in health outcomes and their relationship with temperature and precipitation, or on the health outcomes of specific extreme weather events^3–5^. The first end-to-end health impact attribution study, which estimated the contribution of human-caused climate change to heat-related mortality during the 2003 European heat wave, was conducted in 2016^6^. Over the past decade, at least 20 studies have estimated the present-day health impacts of human-caused climate change (Fig. 1; see the Methods for search criteria).

With a single exception^7^, every health impact attribution study so far has reported a substantial negative health impact of climate change—most often, loss of life due to rising temperatures or extreme weather. Estimates of community-level mortality have ranged from 10 deaths (on a single day of the 2006 heat wave in London^8^) to 1,683 deaths (associated with heat in Zurich between 1969 and 2018^9^); at the national level and above, mortality estimates have ranged from 370 deaths (associated with the summer 2022 heat wave in Switzerland^10^) to 271,656 deaths (associated with heat across 43 countries between 1991 and 2018^11^) (Extended Data Table 1). So far, research effort has been heavily biased towards temperature-related risks (*n* = 17 of 20), mortality (*n* = 11 of 20) and extreme weather events in Europe (*n* = 4 of 6). However, these studies have diversified over time, with recent research addressing the growing burden of mosquito-borne viral diseases^12,13^, mortality due to air pollution from wildfires^14^, population displacement by floods^15^ and several unique health risks experienced by children^16^, including neonatal deaths^17,18^, preterm births (and life-long associations with asthma, type 1 and 2 diabetes and cognitive disabilities)^18^, low birth weight^19^ and childhood malaria^20^.

In some cases, the estimated economic impact or value of these losses has been substantial. For example, one study estimated that medical costs related to heat wave-related preterm births in China could exceed US$300 million per year, and the loss of lifetime earnings associated with cognitive disabilities could exceed US$1 billion per year^18^. Another study estimated that life-years lost due to Hurricane Harvey could be worth roughly US$17 billion^21^, while a global study of 185 extreme weather events estimated an average attributable loss of life valued at US$22.7 billion per year^22^. Here, we apply standard estimates of the value of statistical life (VSL)^23^ to compute monetized losses due to other attributable health impacts. Using both the US Environmental Protection Agency (EPA) VSL and an adjusted version of this figure that accounts for the finding that willingness to pay for reductions in mortality risk varies systematically across countries^23,24^, we estimate that annual losses are also on the order of at least US$10 billion (Methods). For example, we estimate attributable annual adjusted losses equivalent to US$29.5 billion (unadjusted, using the US EPA VSL: US$106.0 billion) due to temperature-related neonatal deaths in 29 low- and middle-income countries^17^; US$31.0 billion (unadjusted: US$111.6 billion) due to heat-related deaths across 43 countries^11^; and US$40.2 billion (unadjusted: US$144.5 billion) due to global fine particulate matter (PM_2.5_) air pollution from wildfires^14^. In some cases, directly quantified losses could be approaching the trillions (Table 1): for example, Vicedo-Cabrera et al.’s estimate of 271,656 climate change-attributable heat-related deaths across 43 countries between 1991 and 2018 would be equivalent to a loss of US$869.3 billion (unadjusted: US$3.1 trillion)^11^. These kinds of estimates are likely to be increasingly important as countries seek financing for loss and damage resulting from climate change^25^, particularly given that human health impacts dominate estimates of aggregate economic damages from future climate change^26^.

**Table 1:** Major estimates of global cause-specific mortality from climate change

Type	Study (time period)	Cause	Deaths (annual)
Attribution studies	Carlson et al.^20^ (2010–2015)	Malaria	2,366 (95% CI −4,925 to 19,423)
	Newman and Noy^22^ (2000–2019)	Extreme weather (excluding heat waves)	2,560
	Childs et al.^12^ (1995–2014)	Dengue fever	3,925 (95% CI 1,915 to 7,028)
	Vicedo-Cabrera et al.^11^ (1991–2018)	Heat (in 43 countries)	9,702 (95% CI 4,005 to 19,135)
	Park et al.^14^ (2010–2019)	Fire-related air pollution mortality	12,566 (range 3,481 to 30,126)
	All five attribution studies listed above	Total	31,119 deaths per year (VSL: US$99.6 to 357.9 billion)
Projection studies	McMichael et al.^29^ (2000)	Floods	2,000
		Heat	12,000
		Malaria	27,000
		Diarrhoeal disease	47,000
		Malnutrition	77,000
		Total	166,000 deaths per year (VSL: US$531.2 billion to US$1.9 trillion)
	Hales et al.^31^ (2030)	Dengue fever	258 (range 136 to 331)
		Heat (>65 years old)	37,588 (range 26,912 to 48,390)
		Diarrhoeal disease (<15 years old)	48,114 (range 21,097 to 67,702)
		Malaria	60,091 (range 37,608 to 117,001)
		Undernutrition (<5 years old)	95,176 (range −119,807 to 310,156)
		Total	241,227 deaths per year (VSL: US$771.9 billion to US$2.8 trillion)

See the Methods for methods used in the derivation of vector-borne disease mortality estimates.

Another important frontier for health impact attribution is source attribution, a set of methods that quantify the contributions of specific major greenhouse gas emitters to extreme events, long-term warming trends and, in some cases, downstream impacts. For example, a recent preprint^9^ estimated that dozens of heat-related deaths in Switzerland between 1969 and 2018 could be attributed to specific fossil fuel companies, led by Chevron (59 deaths), ExxonMobil (54 deaths) and Saudi Aramco (53 deaths). Using the same VSL approach as above, these estimates would be equivalent to losses of US$188.8 million (unadjusted: US$678.5 million), US$172.8 million (unadjusted: US$621.0 million) and US$169.6 million (unadjusted: US$609.5 million) attributable to each emitter, respectively. Source attribution studies are likely to have a unique relevance to legal actions against emitters and governments, which can be supported by evidence linking anthropogenic greenhouse gas emissions—and, potentially, the specific emissions of the defendant company—to the claimants’ losses^27,28^ (Supplementary Text 1).

Increasingly, these studies represent the strongest available line of evidence regarding the present-day health impacts of climate change. In many cases, they are already substantially more up to date than modelling-based estimates: the only comprehensive estimate of present-day (circa 2000) global mortality and morbidity due to climate change was published 20 years ago^29,30^; in 2014, these estimates were extended from 2030 through 2050^31^. For some health impacts, such as heat- and extreme weather-related mortality, attribution studies have been broadly concordant with these projections (Table 1). In other cases, the divergence has been notable: for example, climate change-attributable mortality related to dengue fever has been an order of magnitude greater than expected, while malaria mortality is estimated to be an order of magnitude lower (Methods). Some major expected sources of mortality have still not been reassessed. For example, one attribution study estimated that every 1 °C of anthropogenic warming has resulted in a 1–2% increase in food insecurity^32^, but no estimate exists of attributable mortality from malnutrition. Similarly, there have been no impact attribution studies focused on diarrhoeal diseases, despite several closely related observational studies^33,34^.

New health impact attribution research continues to be published. As the field of health impact attribution continues to grow, these studies can provide a more comprehensive view of the impacts of climate change on mortality, morbidity, life expectancy and well-being. In particular, future studies could assess the impact of climate change on dozens of infectious diseases; non-communicable diseases, including asthma, cancer, diabetes, heart disease and kidney disease^35^; health impacts of food insecurity, including malnutrition, stunting and direct mortality^32^; and mental health, including anxiety, depression and suicides^36^. Future studies should also explore the uneven impacts of climate change within populations. These analyses are currently rare in the attribution literature, although one study found that women and the elderly accounted for 60% and 90% of attributable heat-related deaths, respectively, with older women experiencing mortality at 1.8 times the rate of the general population^10^.

**Extended Data Fig. 1:**
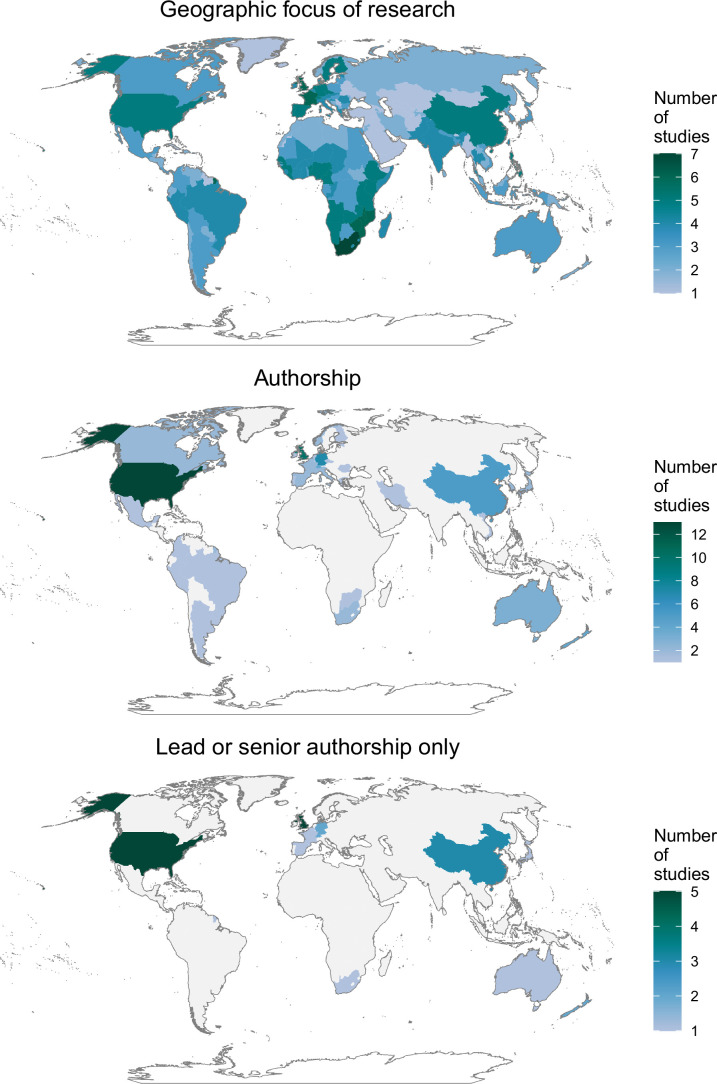
Geography of study scope and author affiliations. Gray values indicate no studies were applicable. (Middle and bottom panel legends indicate the number of studies with at least one author, or lead author, with an affiliation listed in that country).

Finally, future studies should aim to provide a more geographically representative view of the health impacts of climate change. For example, almost all studies at the subnational level have so far focused on communities in high-income countries (*n* = 6 of 7). These biases represent the research community behind these efforts, which are almost entirely led out of global north institutions—even when focused on health impacts in the global south (Extended Data Fig. 1). The authorship dynamics in this subfield are not atypical in global health, which suffers from a deeply inequitable and colonial system for exchanging data, knowledge, scientific credit and international aid^37^. Some researchers have suggested that surfacing more open-access health datasets from governments in the global south will help close this gap^5^, but this is at best a partial solution and at worst will reinforce existing dynamics by making it easier for global north researchers to bypass collaboration altogether. The best way to increase knowledge about the health impacts of climate change is to increase investment in research led by climate scientists and public health researchers living at the frontlines of climate injustice: as one expert recently observed, “knowledge from the global South is in the global South”^38^.

## Methods

### Search strategy

In this study, we focus on studies that conduct end-to-end health impact attribution, which we define as a statistical analysis that quantifies present-day or historical health impacts or risks resulting from anthropogenic (human-caused) forcings on the climate through the comparison of factual and counterfactual scenarios (where the counterfactual scenario usually excludes all anthropogenic forcings). These studies constitute some of the strongest evidence for the health impacts of climate change. However, this is a narrow definition: for example, most health outcomes that are attributable to climate change, based on the broader definition used by the Intergovernmental Panel on Climate Change^39^, have not been identified through the use of an end-to-end impact attribution study.

We used the following keyword set to search for relevant literature on the detection and attribution of human health outcomes to human-caused climate change: (‘climate chang*’ OR ‘climatic change’ OR ‘changing climate’ OR ‘global warming’ OR ‘drought’ OR ‘flood*’ OR ‘storm*’ OR ‘cyclone’ OR ‘extreme weather’ OR ‘monsoon’ OR ‘sea level rise’ OR ‘sea-level rise’ OR ‘heat stress’ OR ‘global heating’) AND (health OR mortality OR morbidity OR ‘infectious disease’ OR ‘non-communicable disease’ OR suicide OR stunting OR miscarriage OR diarrhea OR diarrhoea OR injuries OR cancer OR diabetes OR cardiovascular disease OR stroke OR malnutrition OR malnourish OR anxiety OR depression) AND (attribut* OR counterfactual OR ‘excess mortality’ OR ‘excess cases’ OR DAMIP).

**Extended Data Fig. 2:**
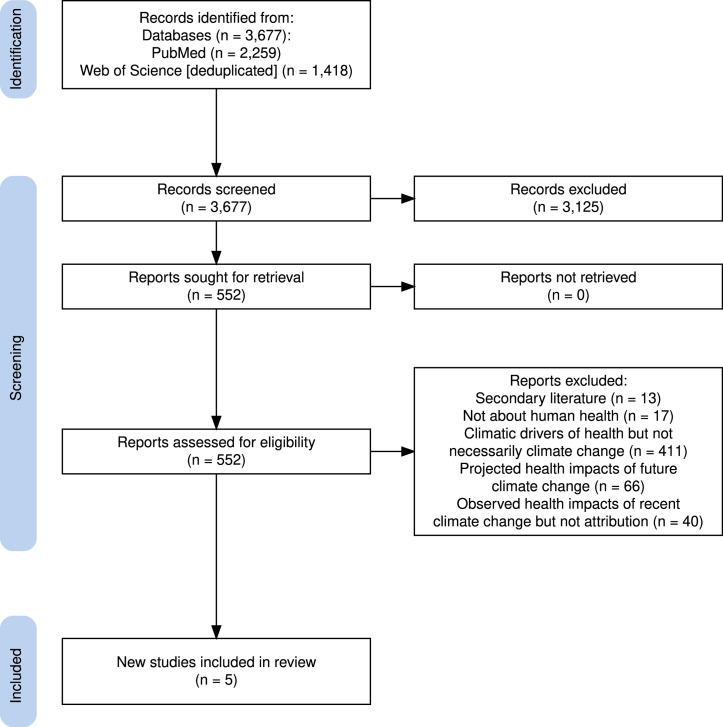
Results of the systematic literature review. The PRISMA template was generated by the PRISMA2020 R Shiny app [ref. ^55^].

We screened PubMed for studies containing these keywords anywhere in the title, abstract or full text (search conducted 21 July 2023) and, to ensure completeness, ran a second search of Web of Science for additional studies containing these keywords in the title or abstract (search conducted 11 September 2023). In total, we screened 3,677 study abstracts for broad relevance to the health impacts of climate change and evaluated the full text of the 552 studies that passed the first round of screening. This led to the identification of only five eligible studies (Extended Data Fig. 2). However, given the lack of standardized language across studies, which we found limited the completeness of the systematic search, we also searched Google Scholar using ad hoc combinations of keywords related to climate change, health and attribution. We also searched five preprint servers (arXiv, medRxiv, bioRxiv, ResearchSquare and SSRN) using the same ad hoc approach. In total, this approach led to the identification of 13 eligible studies in our original systematic search^5^, including one that was originally excluded in a prior version of the search^15^, based on a narrower definition of health impacts.

Since the conclusion of our original systematic literature search, we have continued to monitor Google Scholar and preprint servers for newly published or preprinted studies and have periodically added new studies to an open-access database of health impact attribution literature called the Health Attribution Library (http://healthattribution.org/). Here, we include all seven additional publications and preprints that were posted by 31 December 2024. For completeness, we also note that after this study was concluded, an additional relevant publication from 2024 was identified^40^ that had been overlooked, due to similarities to a preprint already included in the sample^41^.

### Inclusion criteria

We identified a total of 20 peer-reviewed publications and preprints that have conducted end-to-end attribution of human health outcomes to human-caused climate change. These studies all include (1) a statistical model that relates health outcomes to climate variables and (2) an estimate of the health impact of human-caused climate change, isolated through the use of a counterfactual scenario that captures natural climate variability without anthropogenic forcings. We excluded studies that quantified health effects of observed climate change by comparing health outcomes at different points in time (for example, refs. ^42,43^), rather than comparing present-day impacts with a present-day counterfactual that omitted anthropogenic influence on the climate. However, we included one study that compared present-day temperature anomalies, baselined on 1880–1910, with a ‘preindustrial’ climate defined as an anomaly of +0 °C (ref. ^41^). We also excluded a small number of studies that used present-day counterfactual climate scenarios that did not sufficiently distinguish natural and anthropogenic sources of variability^44,45^. Finally, we excluded studies that included a health-related analysis alongside a climate change attribution component, but stopped short of end-to-end attribution of health impacts^46^.

### VSL analyses

Following Newman and Noy^22^, we conducted a secondary analysis of economic losses based on the studies in Extended Data Table 1. Like Newman and Noy, we use the VSL to assess the economic value of deaths attributable to human-caused climate change. Such an approach monetizes lives lost by leveraging estimates of how individuals make their own trade-offs between income and mortality risk. These estimates are recovered using diverse methods, ranging from empirical assessments of how people trade off wages versus mortality risk in the labour market^47^ to stated preference surveys^24^. Resulting calculations reflect the willingness of individuals to pay to avoid the elevated risk of death and are widely used for policy evaluation^48^. However, such a valuation approach does not account for any broader societal economic losses, such as costs to a public healthcare system induced by elevated morbidity or mortality.

To implement this, we follow Newman and Noy in using the US EPA’s value for the VSL, and we treat VSL as equivalent across countries on equity-related grounds. However, we adjust the EPA figure for inflation (US$7.4 million in 2006 = US$11.5 million in 2024 US dollars). We also present two options for how to adjust VSL estimates derived by the EPA for the US population to global populations. This adjustment is motivated by the consistent finding that VSL estimates vary systematically with income, due to lower-income populations having a higher marginal benefit from income (for example, ref. ^23^). In option 1, we adjust for the difference between US and global purchasing power parity-adjusted gross domestic product (GDP) per capita (using World Bank estimates of 2023 GDP adjusted for inflation to 2024 US dollars using the Federal Reserve Economic Data (FRED) GDP deflator^49^ gives a US GDP per capita of US$84,211 versus global average GDP per capita of US$23,403) using an income elasticity of the VSL of 1, reflecting recent economic consensus^48^. This approach reduces our global average VSL estimate to US$3.2 million. In option 2 (which we present in parentheticals throughout), we use the full USA-based value of US$11.5 million everywhere, as Newman and Noy did.

### Estimating climate change-attributable mortality from vector-borne diseases

To supplement the three studies that generated global, cause-specific estimates of climate change-attributable mortality^11,22^, we also extrapolated mortality resulting from two vector-borne diseases, based on preliminary results from two preprints^12,20^. For dengue fever, we used Childs et al.’s estimate that 18% (95% confidence interval (CI) 11% to 27%) of present-day (1995–2014) cases are due to climate change. We multiplied this proportion by an estimated 21,803 (95% CI 17,408 to 26,030) deaths per year due to dengue fever over the same time frame, derived from the Global Burden of Disease study database^50^. For malaria, we used Carlson et al.’s estimate that climate change was responsible for 0.09 (95% CI −0.30 to 0.51) percentage points of the prevalence of malaria in children (ages 2–10) in sub-Saharan Africa between 2010 and 2014, compared with an estimated continent-wide prevalence of 24% in 2015^51^. In the absence of an epidemiological model relating changes in childhood malaria prevalence to age-structured incidence dynamics (and, ideally, accounting for interventions^52^), we assumed that changes in prevalence reflect proportional changes in mortality and used an estimate of 631,000 total deaths (95% CI 394,000 to 914,000) across ages and across the continent in 2015^53^.

## Online content

Any methods, additional references, Nature Portfolio reporting summaries, source data, extended data, supplementary information, acknowledgements, peer review information; details of author contributions and competing interests; and statements of data and code availability are available at 10.1038/s41558-025-02399-7.

## Supplementary information

Supplementary Information Supplementary Text 1. Health impact attribution and litigation.

## Data Availability

All data are publicly available via GitHub at https://github.com/carlsonlab/AttributableLosses and via Zenodo at 10.5281/zenodo.15685967 (ref. ^54^).

## Extended data

**Extended Data Table 1:** Estimates of deaths attributed to anthropogenic climate change

Cause, location, and period	Estimate
Heat-related deaths from the hottest day of the summer 2006 heat wave in London, UK (July 26, 2006)^8^	10 deaths
Deaths due to Hurricane Harvey in Texas, USA (2017) (extrapolated)^21^	51–66 deaths
Heat-related deaths due to the summer 2003 heat wave in Greater London, UK (June 1 to August 31, 2003)^6^	64 deaths (95% CI: 61–67)
*Warm season heat-related deaths during the summer 2018 heat wave in the Canton of Zürich, Switzerland (July 29 to August 10, 2018)^9^	86 deaths (95% CI: 55–113)
Heat-related deaths during the summer 2022 heat wave in Switzerland (June 1 to August 31, 2022)^10^	370 deaths (95% CI: 133–644)
Heat-related deaths due to the summer 2003 heat wave in Central Paris, France (June 1 to August 31, 2003)^6^	506 deaths (95% CI: 455–557)
Neonatal deaths related to preterm births in China (2010 to 2020)^18^	682 deaths (95% CI: 99–902)
Warm season heat-related neonatal death due to pre-term births in China (2010 to 2020)^18^	1,276 deaths (95% CI: 924–1,881)
*Warm season heat-related deaths in the Canton of Zürich, Switzerland (1969 to 2018)^9^	1,683 deaths (95% CI: 270–3,279)
Year-round heat-related childhood deaths in Africa (1995 to 2004)^16^	~3,000 – ~5,000 deaths
Global deaths due to PM2.5 air pollution from fires (1960 to 1969)^14^	6,690 deaths (range: −3,980–17,730)
Year-round heat-related childhood deaths in Africa (2011 to 2020)^16^	~7,000–~11,000 deaths
Global deaths due to 154 extreme weather events (heat and cold waves, floods and droughts, storms, and wildfires) that became more likely due to climate change (2000 to 2019)^22^	75,139 deaths
Global deaths due to PM2.5 air pollution from fires (2010 to 2019)^14^	125,660 deaths (range: 34,810–301,260)
Heat-related neonatal deaths in 29 countries (2001 to 2019)^17^	175,133 deaths (95% CI: 7,806–353,516)
Global warm season heat-related deaths across 732 populations from 43 countries (seasonal average summed over 1991 to 2018)^11^	271,656 deaths (95% CI: 112,140–535,780)

Estimates that are starred are findings from a preprint, and may change in future versions. Only estimates of attributable mortality are shown, and not estimates of deaths averted [refs. ^7,17,22^].

## Code availability

All code is publicly available via GitHub at https://github.com/carlsonlab/AttributableLosses and via Zenodo at 10.5281/zenodo.15685967 (ref. ^54^).
