# Supplementary material for: Health losses attributed to anthropogenic climate change
Source: Nat Clim Chang. 2025 Sep 17;15(10):1052–5. doi: 10.1038/s41558-025-02399-7 (PMC12504110; doi:10.1038/s41558-025-02399-7)
Supplement: Supplementary file 1 — Supplementary Text 1. Health impact attribution and litigation. [file 41558_2025_2399_MOESM1_ESM.pdf]

nature climate change

Supplementary information https://doi.org/10.1038/s41558-025-02399-7

Health losses attributed to anthropogenic climate change

In the format provided by the authors and unedited

Supplementary Material for Carlson et al. (2025)
“Health losses attributed to anthropogenic climate change”

Text S1. Health impact attribution and litigation.

Courts are increasingly hearing cases that aim to hold major emitters liable for their contribution to the impacts of climate change. Health impacts are often at the heart of this litigation – and health impact attribution studies are becoming an important source of potential evidence.

Many of these suits seek financial compensation for high-emitting companies’ contributions to costs incurred by climate change impacts on human health (see e.g., *Luciano Lliuya v. RWE*, where a Peruvian Ministry of Health assessment of flood risk experienced by the plaintiff was cited; or *County of Multnomah v. Exxon Mobil Corp.,* where plaintiffs sought damages for “impacts on public health and infrastructure”). In these lawsuits, claimants may need to demonstrate a causal link between firms’ greenhouse gas emissions and the impacts on plaintiffs, a legal need that may be met through reference to the findings of attribution studies[1]. For example, in May 2024, three NGOs brought a criminal complaint grounded in attribution science that argued that Total Energies’ directors and main shareholders should be held criminally liable for endangering lives on the basis of the company’s contribution to climate change. Cases like these could deliver justice for victims of climate change, and discourage continued harmful conduct if firms are held liable for the costs of their emissions or directors are held personally responsible[2].

Other cases seek accelerated mitigation action. In the 2024 ruling of the European Court of Human Rights in *Verein Klimaseniorinnen Schweiz and others v. Switzerland*, the Court referred to attribution findings in finding evidence of a causal relationship between greenhouse gas emissions and the risk of heat wave-related deaths; this assessment was necessary for the claimants to have standing to bring the case. The Court then ruled that the Swiss government’s climate policy was insufficient to protect the claimant’s rights under the European Convention. Switzerland is now forced to reassess the ambition of their climate policy in light of this ruling — or else, be found to continue to be in breach of the European Convention on Human Rights.

References

1. Stuart-Smith, R. F. *et al.* Filling the evidentiary gap in climate litigation. *Nat. Clim. Chang.* **11**, 651–655 (2021).
2. Wetzer, T., Stuart-Smith, R. & Dibley, A. Climate risk assessments must engage with the law. *Science* **383**, 152–154 (2024).
